# Comprehensive Analysis of Senescence Characteristics Defines a Novel Prognostic Signature to Guide Personalized Treatment for Clear Cell Renal Cell Carcinoma

**DOI:** 10.3389/fimmu.2022.901671

**Published:** 2022-06-02

**Authors:** Peng Zhou, Zheng Liu, Henglong Hu, Yuchao Lu, Jun Xiao, Yanan Wang, Yang Xun, Qidong Xia, Chenqian Liu, Jia Hu, Shaogang Wang

**Affiliations:** Department of Urology, Tongji Hospital, Tongji Medical College, Huazhong University of Science and Technology, Wuhan, China

**Keywords:** cellular senescence, tumor microenvironment, prognostic signature, targeted therapy, immune checkpoint blockade

## Abstract

Accumulating evidence has suggested the impact of senescence on tumor progression, but no report has yet described how senescence shapes the tumor microenvironment of clear cell renal cell carcinoma (ccRCC). The objective of this study was to delineate the senescence features of ccRCC and its role in shaping the tumor microenvironment through a comprehensive analysis of multiple datasets, including 2,072 ccRCC samples. Unsupervised consensus clustering identified three senescence subtypes, and we found that the senescence-activated subtype survived the worst, even in the condition of targeted therapy and immunotherapy. The activated senescence program was correlated to increased genomic instability, unbalanced PBMR1/BAP1 mutations, elevated immune cell infiltration, and enhanced immune inhibitory factors (cancer-associated fibroblasts, immune suppression, immune exclusion, and immune exhaustion signaling). A senescence score based on nine senescence-related genes (i.e., P3H1, PROX1, HJURP, HK3, CDKN1A, AR, VENTX, MAGOHB, and MAP2K6) was identified by adaptive lasso regression and showed robust prognostic predictive power in development and external validation cohorts. Notably, we found that the senescence score was correlated to immune suppression, and the low-score subgroup was predicted to respond to anti–PD-1 therapy, whereas the high-score subgroup was predicted to respond to Sunitinib/Everolimus treatment. Collectively, senescence acted as an active cancer hallmark of ccRCC, shaped the immune microenvironment, and profoundly affected tumor prognosis and drug treatment response.

## Introduction

Renal cell carcinoma (RCC) is a common malignancy of the urinary tract, and its predominant histological type is clear cell carcinoma (80%–90% of cases). Approximately one-third of patients with RCC harbor distant metastases at the first diagnosis, and the 5-year overall survival (OS) of these patients is only 10%–20% versus 70% of patients with localized tumors (1). In recent years, the description of novel cancer subtypes based on expression profiles has helped us gain deeper insight into the molecular features and oncological heterogeneity of RCC, such as the mRNA/miRNA subtypes identified by The Cancer Genome Atlas (TCGA) project (2), the four subtypes of metastatic clear cell renal cell carcinoma (ccRCC) identified to distinguish sunitinib treatment response (3), the seven subtypes to describe the clonal origin of RCC (4), and the seven molecular subtypes of high-grade ccRCC (5). However, these molecular subtypes still face great challenges in differentiating patients’ prognosis and guiding personalized treatment options for ccRCC.

It is well known that RCC is chemotherapy and radiotherapy-insensitive, and targeted therapies represented by anti-angiogenic tyrosine kinase inhibitors (TKIs) and inhibitors of rapamycin protein (mTOR) are the main post-operative adjuvant treatment strategies for patients with ccRCC (6). Immune checkpoint blockade (ICB) presents a new option for ccRCC patients, with approximately 20%–35% objective response rates and a significant survival benefit over targeted therapy (7–10). The pan-cancer analysis reported a moderate tumor mutation burden (TMB) in ccRCC, whereas approximately 70% of ccRCC are immune cells infiltrated, giving ccRCC an abundance of pre-existing anti-tumor immunity (11). However, clinical trials in RCC did not report the predictive value of TMB, baseline CD8+ T infiltration, or PD-1/PD-L1 expression levels for ICB treatment response (10). In addition, several clinical trials testing the efficacy of the ICB+anti-vascular treatment strategy in patients with metastatic ccRCC have reached their endpoints. According to two meta-analyses of published reports, the combination regimen can increase CR rates by more than threefold when compared to anti-vascular monotherapy (12). Therefore, there is an urgent need to introduce novel theories to further elucidate the molecular heterogeneity of ccRCC and to guide personalized treatment selection.

Accumulating evidence has suggested the impact of senescence on tumor progression (13, 14), but no report has yet described how senescence shapes the tumor microenvironment of ccRCC. The current perspective suggests that senescence and tumorogenesis are two different manifestations resulting from the accumulation of cell damage over time. López-Otín et al. summarized and proposed nine biological features to define the senescence phenotype (15). The evolution of the senescence microenvironment is inextricably linked to a shift in fibroblast behavior (13). In the young tissue microenvironment, fibroblasts promote immune infiltration and clearance of adverse factors, whereas, in the senescent microenvironment, fibroblasts undergo a persistent senescence-associated secretory phenotype (SASP) and turn to support immunosuppressive cell infiltration such as MDSC and Treg (16–19). On the other hand, the accumulation of cellular senescence features in macrophages, dendritic cells (DCs), natural killer (NK) cells, and effector T cells also leads to a decrease in anti-tumor immunity (13). In addition, it has been demonstrated that integrity loss of extracellular matrix (ECM) in the senescent microenvironment is another leading cause of the onset and progression of malignant events (20). In other words, the development of the senescence theory may provide new ideas to further delineate the immune characteristics of ccRCC. Therefore, there is a need for understanding the senescence features of ccRCC, especially the association of senescence factors with clinical prognosis and therapeutic benefit, which may be informative for the development of interventions targeting senescence.

Here, we conducted a comprehensive delineation of senescence features in 2,072 ccRCC samples and identified three senescence subtypes with significantly different molecular and immune microenvironmental characteristics. We constructed a senescence score using the senescence-related genes, which is not only a robust prognostic indicator, but subgroup analysis revealed senescence score subgroups with different sensitivity to targeted therapies and immunotherapy.

## Materials and Methods

### Raw Data Retrieval and Preparation

We retrospectively collected expression profiles of frozen tumor specimens from 13 publicly available ccRCC cohorts: RNA-seq data of the TCG-Kidney renal clear cell carcinoma (KIRC) cohort from the TCGA project; a total of nine microarray cohorts based on the GPL570 and GPL10588 platforms archived in the GEO portal (https://www.ncbi.nlm.nih.gov/geo/); and two microarray archived in the ArrayExpress database (https://www.ebi.ac.uk/arrayexpress/). Complete clinical information is available in the TCGA-KIRC, E-MTAB-1980, E-MTAB-3267, and Checkmate, with E-MTAB-3267 containing 53 advanced metastatic ccRCC treated with sunitinib and Checkmate containing 181 advanced ccRCC treated with Nivolumab and 130 patients treated with Everolimus. Baseline information for datasets involved in this study was summarized in **Table S1**. FPKM values of TCGA-KIRC provided by the UCSC Xena website (https://xena.ucsc.edu/) were transformed into TPM values and log2-transformed to maintain comparability with microarray platform-produced data. Raw data of the “CEL” format generated from the Affymetrix platform was background corrected and normalized using the “affy” package. For datasets from the GEO database, six datasets based on the GPL570 platform (i.e., GSE36895, GSE53757, GSE66272, GSE73731, GSE46699, and GSE22541) and three datasets based on GPL10588 (i.e., GSE40435, GSE105261, an GSE65615) were merged as GPL570-merge and GPL10588-merge using the “sva” function of the “Combat” package.

### Senescence Signatures and Cellular Senescence-Related Genes Retrieval

Fifteen senescence-related gene sets were retrieved and archived in the MSigDB database, including nine GO biological processes and six Reactome hallmarks. A total of 279 experimentally confirmed cellular senescence-related genes were archived in the CellAge database (https://genomics.senescence.info/cells/).

### Differentially Expressed Senescence-Related Genes (DESG) Analysis and Functional Annotation

Differential expression analysis was performed using the “limma” package, and an adjusted p-value < 0.05 was set to identify the DESGs. Gene set enrichment analysis (GSEA) was performed using the “ReactomePA” package. GO/KEGG annotation of the DESGs was performed using the “ClusterProfiler” package.

### Identification of the Senescence Subtypes in ccRCC

Gene set variation analysis (GSVA) was developed to estimate the signaling pathway activity of a single sample based on reference gene sets {Haenzelmann:2013ga}. We used the “GSVA” package to estimate the activity of the senescence-related biological processes of tumor samples. The unsupervised consensus clustering using the “km” algorithm was adopted to identify senescence subtypes in tumor samples of TCGA-KIRC based on the GSVA enrichment score. The similarity of samples was determined by the Euclidean distance. One thousand iterations were recycled in the clustering to ensure the stability of the results. The discrimination of the samples of subtypes was assessed using t-distributed stochastic neighbor embedding (t-SNE) downscaling analysis provided by the “Rtsne” package.

### Cancer Hallmarks and Tumor Microenvironment Characteristics Analysis

We use the “IOBR” package to analyze the molecular and immune microenvironment characteristics of ccRCC (21). The cancer hallmarks, GO/KEGG biological process, metabolic, and tumor microenvironment signatures, as well as eight immune cell estimation methods (i.e., Cibersort, MCP, xCell, EPIC, Estimate, quantiseq, IPS, and TIMER) were integrated into the “IOBR” package. We estimated the biological process activity of tumor samples using the calculate_sig_score function. The percentage of immune cells was deconvoluted using the deconvo_tme function. In addition, the relative infiltration abundance of 28 immune cells (16 adaptive immune cells and 12 intrinsic immune cells) was estimated using the ssGSEA algorithm provided by the “GSVA” package (22).

### Construction and Verification of the Senescence Score and Nomogram for Clinical Application

The TCGA-KIRC (n = 514) was used as the development cohort and the E-MTAB-1980 (n = 100) as the test cohort to validate the prognostic model, and the baseline clinic parameters were summarized in **Table S2**. The predictive value of the senescence score for targeted therapy and immunotherapy responsiveness was verified in the E-MTAB-3267 and Checkmate cohorts. To ensure the robustness of the prognostic model, we excluded samples with survival times shorter than 1 month from the TCGA-KIRC and E-MTAB1980 cohorts. Firstly, the Pearson’s correlation of all differentially expressed senescence-related genes with PD-1/PD-L1/CTLA4 expression levels was calculated, and genes with a Bonferroni corrected p-value < 0.05 were retained. Secondly, univariate cox analysis identified prognostic senescence-related genes with p<0.05. Subsequently, the best combination of senescence genes to construct a multivariate cox model was determined by performing adaptive lasso regression using the “glmnet” package. Adaptive lasso regression eliminates the overfitting bias by introducing weights to the traditional lasso model and obtains a more succinct model without compromising model performance (23). A multivariate Cox model was constructed using the senescence genes identified by lasso Cox regression, the coefficient value of each gene was derived, and the senescence score was generated by multiplying the gene expression by the non-zero coefficient of each gene:


Senescence score=∑Gi∗Bi

where Gi is the expression level and Bi is the coefficient. A nomogram was built up using the “rms” package and assessed by the calibration curves.

### Prediction of Patients’ Sensitivity to Targeted Therapy and Immunotherapy

We used the “pRRophetic” package to infer the sensitivity of tumor samples to targeted therapeutic agents. Specifically, the cancer cell gene expression profiles and corresponding IC50 values under drug agent treatment retrieved from the GDSC database were used as references. pRRophetic runs a 10-fold cross-validation ridge regression to estimate the IC50 values of the ccRCC samples based on their gene expression profiles. The similarity of gene expression profiles between the ccRCC samples and 47 skin melanoma patients treated with anti-CTLA4/PD-1 was inferred using the Subclass Mapping module provided by the GenePattern portal (24, 25).

### Statistical Analysis

All statistical analysis was performed and all results were visualized in Rstudio 4.0.1. The Wilcoxon or Kruskal–Wallis tests evaluated the difference between two or more groups of continuously distributed variables. Fisher’s exact test evaluated the distribution difference of categorical variables. Survival status was visualized by the Kaplan–Meier method, and the OS difference was assessed using the log-rank test. The ROC curves were adopted to assess the predictive capacity of the prognostic indicators. The Bonferroni correction for multiple tests was adopted to reduce the probability of Class I error, and the statistical difference was considered significant when the p-value of bilateral tests < 0.05.

## Results

### Senescence Signatures Are Generally Activated in ccRCC

**Figure 1:**
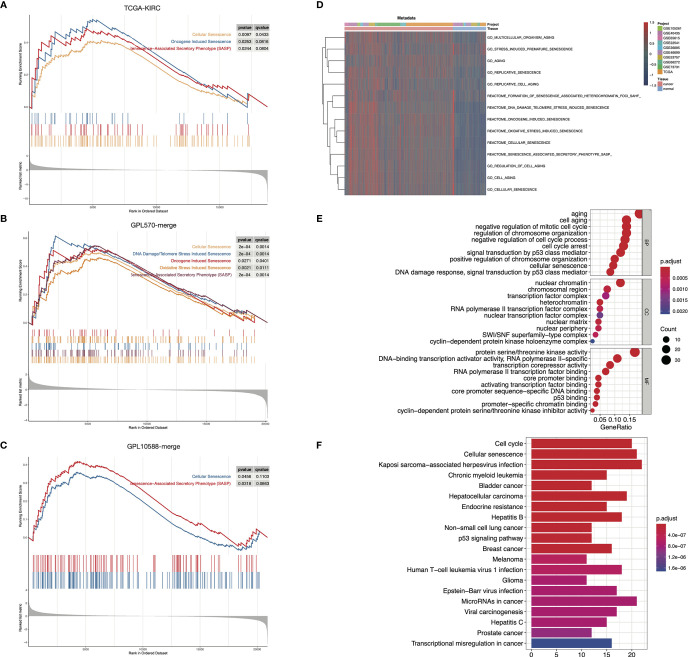
Senescence-related biological processes were highly activated in ccRCC. **(A–C)** GSEA results showed that senescence-related biological processes were activated in ccRCC tumor samples in TCGA-KIRC **(A)**, GPL570-merge **(B)**, and GPL10588-merge **(C)** datasets. **(D)** Senescence-related biological processes were generally activated in ccRCC tumor samples in the metadata set. The biological process activity was assessed using the “GSVA” package. **(E, F)** Functional annotation of the 204 DESGs in the GO **(E)** and KEGG **(F)** database.

To delineate the senescence characteristics of ccRCC, we conducted a comparison of tumor and para-cancerous tissues in three independent datasets. GSEA analysis of TCGA-KIRC showed that cellular senescence, oncogene-induced senescence, and SASP were significantly activated in tumor samples (**Figure 1A**). Similar results were obtained in the GPL570-merge and GPL10588-merge datasets (**Figures 1B, C**). We merged the three datasets into metadata, and the heatmap showed that the senescence-related GO/Reactome terms were markedly activated in tumor samples (**Figure 1D**). These results suggest that senescence is an important hallmark of ccRCC. Using DEG analysis, we identified 204 differentially expressed senescence-related genes in TCGA-KIRC, including 124 upregulated and 82 downregulated (**Table S3**). GO annotation showed the significant enrichment of cell aging, regulation of the cell cycle, and chromosomal behavior (**Figure 1E**). The KEGG annotation showed the enrichment of the cell cycle, cell senescence, and p53 signaling pathway (**Figure 1F**).

### Senescence Subtypes Identification in ccRCC Based on Senescence Signatures

**Figure 2:**
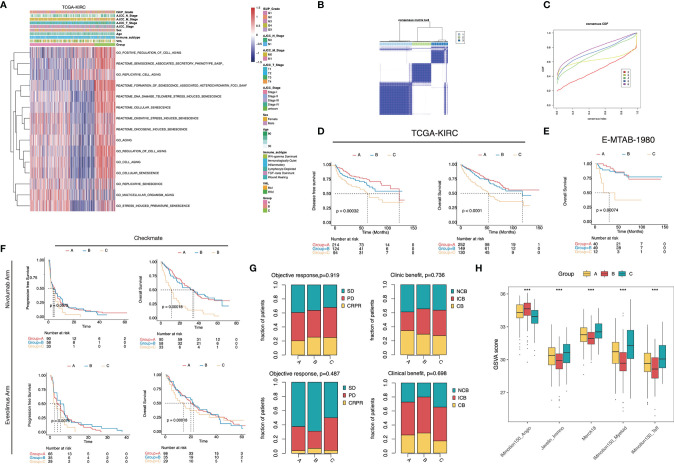
Senescence subtype was correlated with stratified patients’ prognosis and treatment benefits. **(A)** Heatmap of the 15 senescence-related biological processes of the senescence subtypes in TCGA-KIRC. **(B)** The heatmap of the k-means consensus clustering result. **(C)** CDF curves of the consensus clustering. **(D–F)** Kaplan–Meier curves showed the survival difference among the senescence subtypes in TCGA-KIRC **(D)**, E-MTAB-1980 **(E)**, and Checkmate **(F)**. Log-rank test. **(G)** Bar plots displayed the proportion of objective response and clinical benefit rates among the senescence subtypes in Checkmate. **(H)** Boxplot showed the difference of the VEGF- and inflammatory-related signature scores in Checkmate. ***, p<0.001.

To better understand the senescence phenotypes in ccRCC, we performed unsupervised consensus clustering based on the activity of the senescence-related GO/Reactome terms. The clustering heatmaps and cumulative distribution function (CDF) curves suggested the existence of three senescence subtypes in ccRCC (**Figures 2A-C**). The t-SNE plots exhibited good discrimination among the three clusters (**Figure S1B**). We termed them senescence-silenced (Cluster A), senescence-suppressed (Cluster B), and senescence-activated phenotypes (Cluster C), respectively (**Figure 2A**). The heatmap of the DEG showed that senescence-related genes were generally inhibited in senescence suppressed phenotype while highly expressed in senescence activated phenotype (**Figure S1A**). The OS and disease-free survival (DFS) rates of the senescence-activated phenotype were significantly lower than the senescence-suppressed and senescence-silenced phenotypes (**Figure 2D**). We then generated three senescence subtypes in the E-MTAB-1980 and Checkmate cohorts for further analysis (**Figures S1C–F**). Similarly, patients of the senescence-activated phenotype in E-MTAB-1980 survived the worst (**Figure 2E**). Interestingly, the senescence-activated subtype had the worst progression-free survival (PFS) and OS rates in both the Nivolumab- and Everolimus-treated arms (**Figure 2F**). Whereas no significant differences in objective response or clinical benefit were found among the subtypes (**Figure 2G**). The correlation of previously published VEGF pathway- and inflammation-related gene signatures with senescence subtypes was also investigated. The results showed an increase in immunoinflammatory-related gene signature activity (Javelin_Immuno, Merck18, IMmotion150_Myeloid, IMmotion150_Teff), and a decrease in VEGF-related gene signature activity (IMmotio150_Angio) as the senescence program was activated (**Figure 2H**).

### Molecular and Immune Microenvironment Characteristics of the Senescence Subtypes

**Figure 3:**
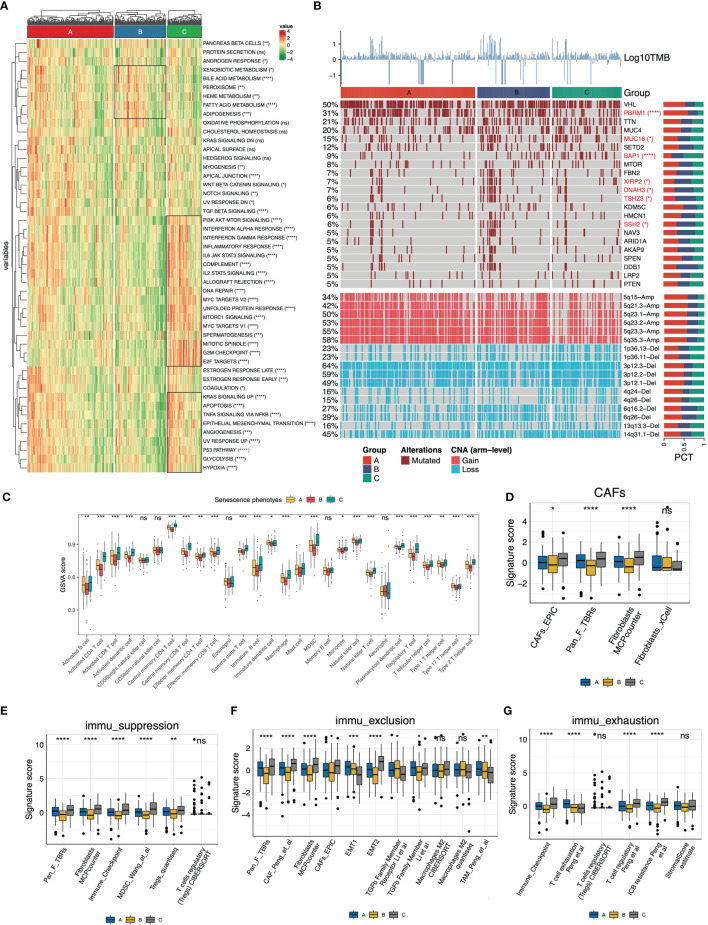
Molecular and immune features of the senescence subtypes of TCGA-KIRC. **(A)** Heatmap of the cancer hallmarks estimated by the ssGSEA algorithm. **(B)** Genomic heterogeneity of the senescence subtypes. The heatmap displayed log10TMB, gene mutation profile (frequency >5%), and differentially distributed arm-level copy number variants (CNV) from top to bottom, respectively. The distribution difference was determined by Fisher’s exact test. **(C)** The 28 immune cells (16 adaptive and 12 intrinsic immune cells) were differentially distributed in the senescence subtypes. The infiltration abundance was estimated by the ssGSEA algorithm. **(D–G)** Signature panels calculated using the “IOBR” package, **(D)** Cancer-associated fibroblasts (CAFs), **(E)** immune suppression, **(F)** immune exclusion, and **(G)** immune exhaustion signatures. The level of statistical difference was labeled with ns, *, **, ***, and ****, which represents no statistical difference, p < 0.05, p < 0.01, p < 0.001, and p < 0.0001, respectively.

We then depicted the cancer hallmarks of senescence subtypes. The results showed that the majority of cancer hallmarks were significantly differentially distributed among the subtypes (**Figure 3A**). The activity of cell cycle regulation-related biological processes such as DNA repair, MYC targets, and G2M checkpoints was highest in the senescence-activated subtype. The senescence-suppressed subtype was associated with metabolic pathways such as adipogenesis, bile acid metabolism, and fatty acid metabolism.

In terms of genomic heterogeneity, we focused on the differentially distributed mutations and copy number variants (CNV). The upper panel of the heatmap (**Figure 3B**) displayed the mutation spectrum of genes with frequencies >5%. We identified six differentially distributed mutated genes (PBRM1, MUC16, BAP1, XIRP2, DNAH3, TSHZ3, and SSH2). The senescence-activated subtype carried fewer PBRM1 mutations and more BAP1 mutations. The bottom panel of the heatmap (**Figure 3B**) displayed the differentially distributed chromosomal fragment copy number alterations, with an overall decreased copy number amplification and increased deletion events in the senescence-activated subtype. More specifically, CNV in the senescent subtype is characterized by decreased 5q fragment amplification and increased 3p fragment deletion.

Subsequently, we deconvoluted the tumor microenvironment of the senescence subtypes. Overall, the abundance of immune cell infiltration, except for NK cells, eosinophils, and neutrophils, within the tumor tended to increase with senescence program activation (**Figure 3C**), which was consistent with the increased immunoinflammatory-related gene signature activity in the Checkmate cohort in **Figure 2H**. Meanwhile, we checked the signaling activity of cancer-associated fibroblasts (CAFs), immune suppression, immune exclusion, and immune exhaustion (**Figures 3D–G**). Dramatically, we found these features were also significantly upregulated along with senescence activation. Signatures from different gene expression profiles suggest that the senescence program is closely correlated with CAFs of the tumor stroma and self-limited antitumor immunity.

### Development of Senescence Score to Predict Patients’ Prognosis

**Figure 4:**
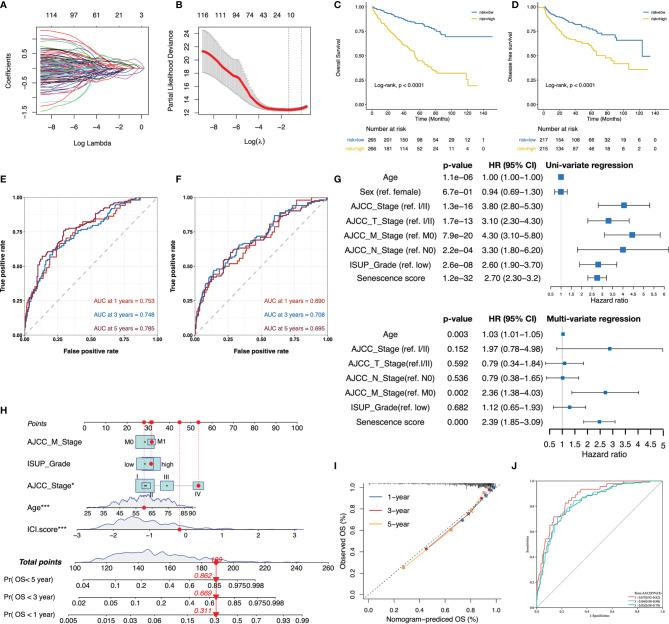
The senescence score was a prognostic indicator for patients’ prognosis in TCGA-KIRC. **(A, B)** The adaptive lasso regression selected the best combination of senescence genes to construct a multivariate Cox model. **(C, D)** Survival analysis showed a different survival portion between the high- and low- senescence score subgroups in TCGA-KIRC for OS **(C)** and DFS **(D)**. **(E, F)** The predictive power of the senescence score for 1-, 3-, and 5-year OS **(E)** and DFS **(F)** was assessed by ROC curves. **(G)** Forest plots of the uni- and multivariate Cox models in TCGA. **(H)** Nomogram to predict patients’ overall survival. The model incorporated the AJCC_Stage, ISUP_Grade, metastatic status, patients’ age, and the senescence score. **(I)** The calibration curves show that the survival status of patients predicted by nomogram at 1, 3, and 5 years remains highly consistent with that actually observed. **(J)** ROC curves to evaluate the predictive efficacy of the nomogram for overall survival at 1, 3, and 5 years. *,p<0.05, ***, p<0.001.

The senescence subtypes profoundly influence tumor progression and the immune landscape, leading to distinctive clinical outcomes. Here, we developed a multivariate model containing nine senescence-related genes (i.e., P3H1, PROX1, HJURP, HK3, CDKN1A, AR, VENTX, MAGOHB, and MAP2K6; **Table S4**) by performing adaptive lasso regression to predict patients’ outcomes (**Figures 4A, B**). The senescence score was generated by multiplying the gene expression by the corresponding non-zero coefficient (**Table S4**). Patients were stratified into two groups using the median senescence score, and significantly poorer survival rates were observed in the high- senescence score group (**Figures 4C, D**). The predictive accuracy of senescence scores at 1, 3, and 5 years for OS was 0.753, 0.748, and 0.785, respectively, whereas it was 0.690, 0.708, and 0.695 for DFS, respectively (**Figures 4E, F**). The senescence score remained an independent unfavorable factor for patients’ prognosis after adjusting clinicopathological parameters (**Figure 4G**). To facilitate clinical application, we integrated the senescence score with patients’ age, American Joint Committee on Cancer (AJCC) stage, metastatic status, and international summer university program (ISUP) grade to establish a nomogram (**Figure 4H**). The calibration curves for the nomogram were plotted, and the results showed that the actually observed status was very close to the predicted survival status at the observation time points, indicating a robust predictive capacity of the nomogram (**Figure 4I**). ROC curves assessed the predictive power of the senescence score for patient’s prognosis at 1, 3, and 5 years, and the areas under curves (AUC) reached 0.87, 0.84, and 0.83, respectively (**Figure 4J**).

**Figure 5:**
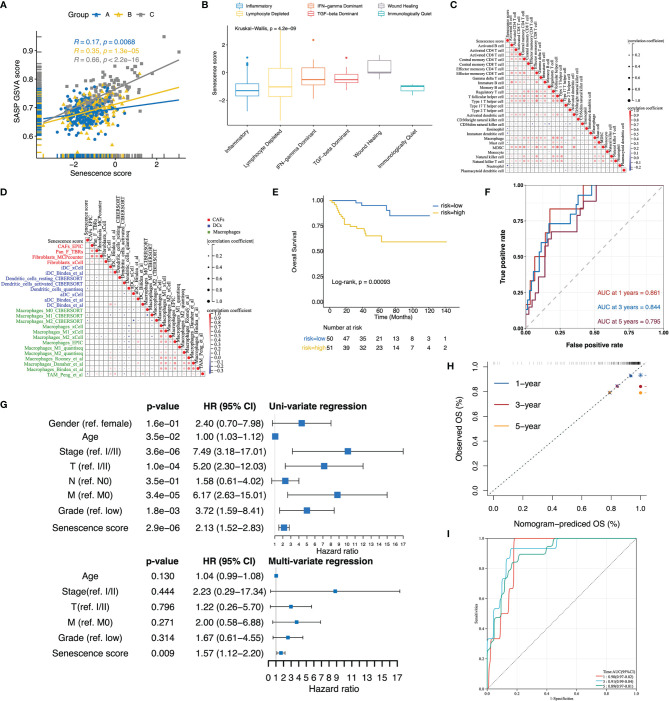
Validation of the senescence score and nomogram to predict patients’ overall survival in E-MTAB-1980. **(A)** Scatterplot with regression lines for senescence score with SASP activity stratified by senescence subtypes. **(B)** The boxplot displayed that the senescence score was differentially distributed among immune subtypes. **(C, D)** Correlation plots displayed a negative correlation of the senescence score with infiltrated immune cells **(C)**, CAFs, DCs, and macrophage signatures **(D)**. The correlation was evaluated using Pearson’s correlation coefficients. **(E)** In E-MTAB-1980, the Kaplan–Meier curves revealed a different survival portion between the high- and low-senescence score subgroups. **(F)** ROC curve assessed the predictive power of the senescence score for 1-, 3-, and 5- year overall survival in E-MTAB-1980. **(G)** Forest plots of the uni- and multivariate Cox models in E-MTAB-1980. **(H)** The calibration curves showed that the survival status of patients predicted by the nomogram applied to E-MTAB1980 at 1, 3, and 5 years remains highly consistent with that actually observed. **(I)** ROC curves to evaluate the predictive efficacy of the nomogram for overall patient survival at 1, 3, and 5 years in E-MTAB-1980.

Given the SASP phenotype mediates the transition in cancer-inhibiting to cancer-promoting roles of the senescence process, we next assessed the relationship between the senescence score and SASP activity. The senescence score was positively correlated to the SASP score in each subtype, and the highest Pearson’s coefficient was detected in the senescence-activation group (**Figure 5A**). In terms of immunophenotypes, wound healing and TGF-dominant phenotypes had the highest senescence scores, whereas inflammatory and immunologically quiet phenotypes had the lowest (**Figure 5B**). Subsequently, we found that the senescence score was negatively correlated to the immune cell infiltration levels by performing correlation analysis (**Figure 5C**). Furthermore, the senescence score was negatively correlated with CAFs and antigen-presenting cells (DCs and macrophages) as quantified by several deconvolution tools (**Figure 5D**). These results suggest that high senescence scores suppress the anti-tumor immune potential.

### Verification of the Senescence Score and Nomogram in Predicting Patients’ Prognosis

The predictive value of the senescence score for the patients’ prognosis was further tested in E-MTAB-1980. Similar to TCGA-KIRC, a comparison of subgroups based on cohort-specific median senescence scores demonstrated significantly decreased OS rates in the high-score group (**Figure 5E**). The senescence score achieved a predictive efficacy of 0.861, 0.844, and 0.795 for 1-, 3-, and 5-year survival, respectively (**Figure 5F**). Even after adjusting for patients’ age, clinical stage, T/M stage, and tumor grade, the senescence score remained an independent risk factor for patients’ prognosis (**Figure 5G**). When applying the nomogram established in TCGA-KIRC to E-MTAB1980, the calibration curve exhibited consistency between the nomogram-predicted and actually observed survival status (**Figure 5H**). Furthermore, the nomogram’s predictive efficacy for the patients’ prognosis at 1, 3, and 5 years reached an impressive 0.9, 0.91, and 0.89, respectively (**Figure 5I**).

### Correlation of the High Senescence Score Group With Targeted Therapy Benefit

**Figure 6:**
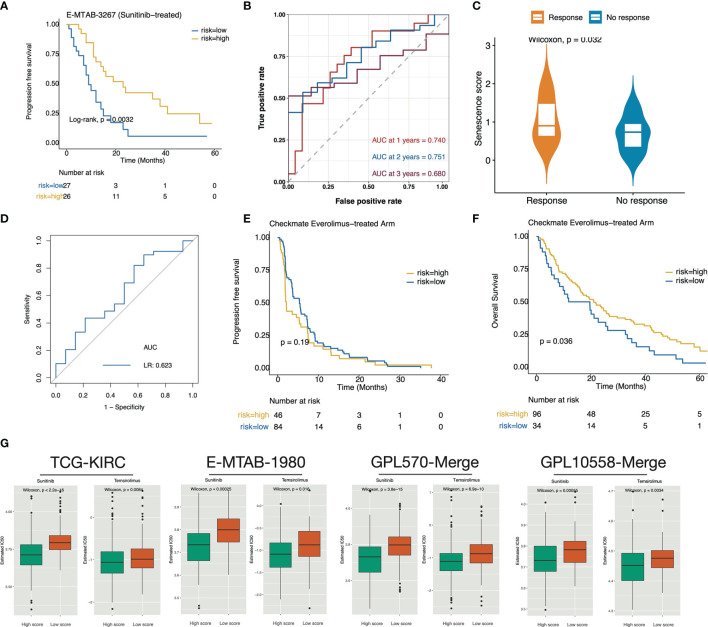
The high senescence score subgroup was correlated with targeted therapy benefit. **(A)** Kaplan–Meier curve exhibited extended PFS time in patients of the high-senescence score groups. **(B)** The predictive value of the senescence score for PFS after sunitinib treatment. **(C)** Boxplot showed that the Sunitinib-response group possesses significantly higher senescence scores. **(D)** Predictive power of the senescence score for sunitinib response. **(E, F)** Survival curves showed that the high-score group determined by the surv_cutponit function gained OS benefit **(F)** in Everolimus treatment rather than PFS benefit **(E)**. **(G)** The predicted IC50 values for Sunitinib and Temsirolimus in the TCGA-KIRC, E-MTAB-1980, GPL570-merge, and GPL10588-merge datasets. A lower IC50 value represents more sensitivity to the drug treatment.

Patients in E-METAB-3267 were all treated with sunitinib (n = 53). Validation of E-METAB-3267 showed an extended PFS in the high-senescence score subgroup (**Figure 6A**). The senescence score achieved a predictive efficacy of 0.74, 0.751, and 0.680 for 1-, 2-, and 3-year PFS, respectively (**Figure 6B**). When comparing the Sunitinib-response and no-response groups, we found significantly higher scores in the Sunitinib-response group (**Figure 6C**), with a predictive efficiency of 0.623 for Sunitinib treatment response (**Figure 6D**). Everolimus is an mTOR-targeted inhibitor for patients who have failed Sunitinib or Sorafenib therapy. Testing in the Checkmate-Everolimus treatment arm found no PFS benefit (p = 0.19) but an OS benefit (p = 0.036) in the high-score group (**Figures 6E, F**). In addition, we stratified TCGA-KIRC, E-MTAB-1980, GPL570-merge, and GPL10588-merge by cohort-specific median senescence score and inferred the drug sensitivity to Sunitinib (VEGF-targeted) and Temsirolimus (mTOR-targeted). In the high-senescence score subgroup (**Figure 6G**), we found significantly lower predicted IC50 values for Sunitinib and Temsirolimus. Collectively, these results indicated anti-VEGF/mTOR-based targeted treatment benefits in the high-senescence score subgroup.

### Correlation of the Low Senescence Score Group With ICB Benefit

**Figure 7:**
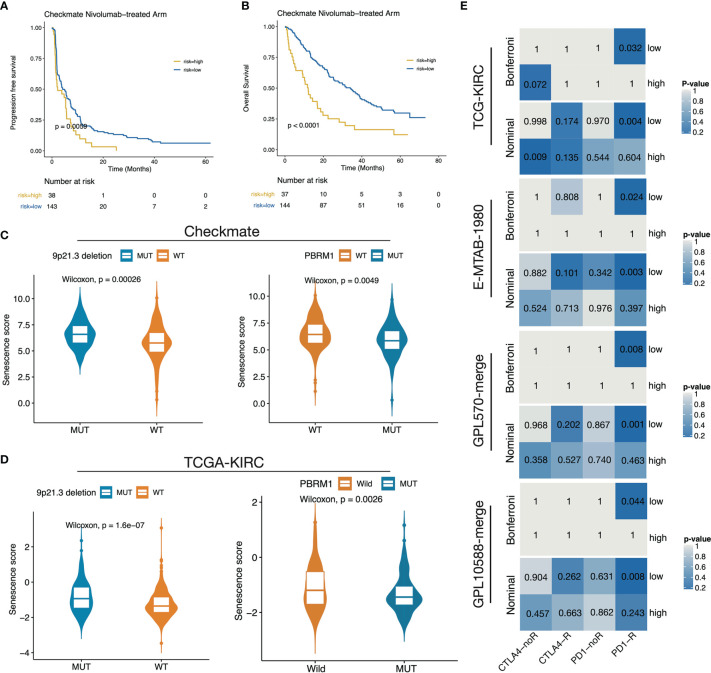
The low senescence score subgroup was predicted to respond to anti–PD-1 treatment. **(A, B)** Survival curves showed that the high-score group determined by the surv_cutponit function gained PFS **(A)** and OS **(B)** benefits. **(C, D)** The boxplot showed that 9p21.3 loss groups possess higher senescence scores while PRBM1 mutant groups possess lower senescence scores in Checkmate **(C)** and TCGA-KIRC **(D)**. **(E)** Subclass mapping results in TCGA-KIRC, E-MTAB-1980, GPL570-merge, and GPL10588-merge datasets using a melanoma dataset treated with anti-CTLA4/PD-1 as a reference.

We next investigated the association of the senescence score with immunotherapy benefit in the Checkmate cohort. We classified patients by median senescence score and found a significant OS benefit of Nivolumab treatment over Everolimus treatment in the low-senescence score subgroup (**Figures S2A, B**). In contrast, no significant PFS/OS survival difference between the treatment arms was observed in the high-senescence score group (**Figures S2C, D**). For Nivolumab treatment, patients in the high-senescence score group determined by the surv_cutpoint function had significantly shorter PFS and OS time (**Figures 7A, B**). Recently, 9p21.3 deletion was reported as an unfavorable factor for immunotherapy responsive in CD8+ T infiltrated ccRCC, whereas PBRM1 mutation was a favorable factor in non-infiltrated ccRCC (10). We observed significantly higher senescence scores in the 9p21.3 loss group and lower scores in the PBRM1 mutation group in both the Checkmate and TCGA-KIRC cohorts (**Figures 7C, D**). In the context of senescence score suppressing immune infiltration, these findings further explain the strong association of the high senescence score group with ICB treatment resistance. Finally, we performed submap mapping inference to further validate the association of the senescence score with ICB benefit in four independent cohorts. As a result, the four cohorts consistently showed a high concordance of the gene expression profiles between low-score group patients and anti–PD-1 SKCM responders (**Figure 7E**), demonstrating the low senescence score group would benefit from anti–PD-1 treatment.

## Discussion

In this study, our exploration of multiple independent ccRCC datasets revealed that activation of senescence-related biological processes is a hallmark of ccRCC. Through delineating the three senescence subtypes, we found that the senescence-activated subtypes possess the worst oncological outcomes, even in the condition of targeted therapy/immunotherapy. Cellular senescence is defined as permanent cell cycle arrest, in which p53/CDKN1A and CDKN2A/pRB signaling play a leading role (14). We found that cell cycle–related signals, including G2M checkpoint, p53 signaling, MYC target, and accumulation of cellular damage events, such as DNA repair and apoptosis, are enriched in the senescence-activated subtype in ccRCC. In addition, senescence program activation was also correlated to the activation of tumor malignant events such as hypoxia, angiogenesis, and EMT signaling.

ccRCC is characterized by widespread loss of 3p and amplification of 5q fragments, and genes encoded by 3p fragments (e.g., PBRM1, BAP1, and SETD2) are frequently mutated and closely correlated to altered prognosis (26). Both BAP1 and PBRM1 are encoded in the 3p21.1 location and are involved in chromatin remodeling to maintain genomic stability. We observed that the senescence subtypes are closely associated with 3p loss/5q amplification and differentially distributed PBMR1/BAP1 mutations, indicating a non-negligible role in ccRCC progression that the senescence process plays. The PBRM1 mutation has been linked to activated angiogenesis and improved sunitinib treatment outcomes when compared to Atezolizumab+Bevacizumab combination treatment (5). In addition, the PBRM1 mutant subgroup also conferred a significant benefit compared with the wild-type subgroup in the Sunitinib treatment arm (27). Clinical trials also reported the correlation of PBRM1 mutation with improved Everolimus treatment outcomes (28), which was demonstrated to be a result of mTOR signaling activation (26). Interestingly, the most recent clinical trial reported the correlation of PBRM1 mutation with anti–PD-1 treatment response in CD8+ T no-infiltrated ccRCC (10). The BAP1 mutation is not only an independent risk factor for ccRCC prognosis (29) but is also linked to poorer Sunitinib/Everolimus treatment outcomes (28). The unbalanced distribution of PBRM1/BAP1 mutations provided a genomic-level explanation for the association of the senescence-activated subtype with poor targeted/immunotherapy treatment outcomes.

Literature also demonstrated that targeted therapeutic agents were able to induce the senescent phenotype of kidney cancer cells. Zhu et al. reported that Sunitinib-treated RCC cell lines manifested distinct senescence features such as the SASP phenotype and cell cycle arrest with DNA damage. This work suggests that the benefit of sunitinib treatment may be attributed to p53/Dec1 signaling activation and drug-induced cellular senescence (30). Another study showed that Axitinib induced DNA damage response in a ROS-dependent manner and eventually led to G2M cell cycle arrest and a senescent phenotype in RCC cell lines (31). Similarly, Mongiardi et al. reported that Axitinib could trigger cellular senescence through oxidative stress-dependent activation of the ATM kinase (32). In summary, these facts provide a theoretical basis for our finding that the high senescence score subgroup would benefit from targeted therapy. Moreover, given that TKI treatment can induce cancer cellular senescence, combining TKI with senescent cell scavengers might be an option for the development of novel treatment strategies for ccRCC. The proposal has already shown promising preliminary results in some preclinical studies, such as the combination of MDM2 inhibitors with AURKA inhibitors (senescence inducers), which not only induced melanoma cell death but also promoted the expression of numerous immune factors, thereby enhancing the anti-tumor immune response (33). However, currently known senescence scavengers, such as MDM2 inhibitors and BCL-2 family inhibitors, are not cell specific, and, therefore, the removal of senescent anti-tumor immune cells will lead to unpredictable toxic side effects (34, 35).

This study also represented the first report to deconstruct the intrinsic association between ccRCC senescence and the immune microenvironment features. Overall, the senescence process not only enhanced immune infiltration but, more importantly, promoted multiple immunosuppressive factors, such as CAFs, immune exclusion, and immune exhaustion signaling at the same time, leading to poorer oncologic outcomes. Using the adaptive lasso regression, we established a senescence score and demonstrated it was negatively correlated with immune infiltration. This behavioral shift might be attributed to the duplex impact of the SASP phenotype on the remodeling of the immune microenvironment. Identified SASP factors include soluble signaling molecules (e.g., interleukins, chemokines, inflammatory factors, and growth factors), proteases, and ECM components (17). Actually, it has long been noted that the stromal fibroblasts manipulate the pro-tumoral/anti-tumoral role of senescence through the SASP phenotype-associated secretion profiles (17, 36, 37). For example, MMP-2 and MMP-9, which are mainly secreted by CAFs, have been shown to be associated with RCC progression (38). However, different studies have reported a pro- or anti-tumorigenic ability of CAFs through the secretion of several factors, which is still debated and may be dependent on tumor type, tumor stage, CAF–tumor interaction, and senescence (39, 40). In addition, evidence demonstrated that the senescent secretome could be modified when there is an interaction between the tumor cells and fibroblasts (41). Collectively, it is currently believed that transient SASP is beneficial, whereas chronic SASP leads to negative outcomes. Many members of the SASP factors can promote tumor invasion or be involved in helping tumor cells evade immune clearance (13, 17). The combination of ICB with key SASP factor-targeted therapies, such as NOTCH and TGF-a inhibitors, holds the potential to block or reverse SASP-induced immunosuppression, thereby enhancing anti-tumor response (42). On the other hand, it is unclear how immune cell senescence affects their functional status, specifically M1 macrophages, DC cells, and CD8+ T cells, and whether interventions on key inducers of cellular senescence can rescue exhausted cytotoxic lymphocytes. Nonetheless, we demonstrated that the remodeling effect of senescence on the immune microenvironment leads to different immunotherapeutic outcomes in patients, and the senescence score was able to distinguish anti–PD-1 responders in ccRCC.

Last, as a preliminary exploration, this study can be further improved in some aspects. First, characterization of senescence features in key immune cell populations, such as CAFs, macrophages, and CD8+ T cells, based on single-cell sequencing data would help us better define stable and specific cellular senescence markers. Second, limited by the available open-access data, this study failed to further evaluate the role of senescence-related genes and senescence score in the clinical benefit of ICB plus anti-VEGF therapy. Given that the combination treatment strategy revolutionized the management of metastatic ccRCC, further investigation into this topic is necessary and desirable.

## Conclusion

In summary, this study elucidated that the senescence process is closely correlated to genomic instability and unbalanced PBMR1/BAP1 mutations in ccRCC. The senescence microenvironment, which switches from immune activation to immune suppression, has a significant impact on anti-tumor immunity. We successfully constructed a senescence score, which is not only a robust prognostic indicator for patients’ prognosis but also provides a new reference basis for personalized treatment selection.

## Data Availability Statement

The original contributions presented in the study are included in the article/**Supplementary Material**. Further inquiries can be directed to the corresponding authors.

## Ethics Statement

The data source involved in this present study was open access; therefore, ethics approval was not applicable.

## Author Contributions

SW, JH, and ZL proposed and designed the framework of this study; QX and CL completed the collation and pre-processing of the raw data required for subsequent analysis; PZ, HH, and YL performed a detailed analysis the raw data; JX and YW visualized the analytical results; PZ interpretated the results and drafted this manuscript; YX, ZL, JH, and SW reviewed and critically revised the manuscript. All authors contributed to the article and approved the submitted version.

## Funding

This presented study was supported by the National Natural Science Foundation of China, Youth Science Fund Project (Grant Number: 81772729), Natural Science Foundation of Hubei Province (No. ZRMS2020002466), and the Chen Xiao-ping Foundation for The Development of Science and Technology of Hubei Province (No.202094). The funders have no role in designing and completing this study.

## Conflict of Interest

The authors declare that the research was conducted in the absence of any commercial or financial relationships that could be construed as a potential conflict of interest.

## Publisher’s Note

All claims expressed in this article are solely those of the authors and do not necessarily represent those of their affiliated organizations, or those of the publisher, the editors and the reviewers. Any product that may be evaluated in this article, or claim that may be made by its manufacturer, is not guaranteed or endorsed by the publisher.

## Supplementary Material

Supplementary Figure 1 Regeneration of the senescence subtypes in external cohorts. **(A)** Heatmap displayed the expression level of the DESGs across the senescence subtypes in TCGA-KIRC. **(B, D, F)** the t-SNE down-dimension plots showed good discrimination of tumor samples among the senescence subtypes in TCGA-KIRC **(B)**, E-MTAB-1980 **(D)**, and Checkmate **(F)**. **(C, E)** Heatmaps of the 15 senescence-related biological processes in E-MTAB-1980 **(C)** and Checkmate **(E)**.

Supplementary Figure 2 Subgroup analysis of ICB treatment benefit versus targeted therapy in Checkmate. **(A, B)** In patients of the low-senescence score group, the use of Nivolumab resulted in an OS, but not a PFS benefit over Everolimus. **(C, D)** In the high-senescence score group, there was no PFS or OS difference between the Nivolumab- and Everolimus-treatment arms.

Supplementary Table 1 Information of datasets involved in our study.

Supplementary Table 2 Clinic information for the development and validation datasets of the prognostic model.

Supplementary Table 3 Differentially expressed senescence-related genes in TCGA-KIRC (p < 0.05).

Supplementary Table 4 Features and corresponding coefficients for the generation of the senescence score.

## Abbreviations

RCC, renal cell carcinoma; ccRCC, clear cell renal cell carcinoma; TCGA, The Cancer Genome Atlas; GEO, Gene Expression Omnibus; ICB, immune checkpoint blockade; MDSC, myeloid-derived suppressor cells; CNV, copy number variation; FPKM, fragments per kilobase million; TPM, transcripts per kilobase million; ssGSEA, single-sample gene set enrichment analysis; GSVA, gene set variation analysis; GO, gene ontology; KEGG Kyoto Encyclopedia of Genes and Genomes; DESG, differentially expressed senescence-related genes; TMB, tumor mutation burden; CR, complete recession; PR, partial recession; SD, stable disease; PD, progressed disease; CB, clinical benefit; ICB, intermediate clinical benefit; NCB, no clinical benefit; OS, overall survival; DFS, disease free survival; PFS, progression free survival; TKI, tyrosine kinase inhibitor; MDSC, myeloid-derived suppressor cells; NK, natural killer; DC, dendritic cell; CAF, cancer-associated fibroblast; LASSO, least absolute shrinkage and selection operator; SASP, senescence-associated secretory phenotype; SKCM, skin cutaneous melanoma; ECM, extracellular matrix; ROC, receiver operating characteristic curve; AUC, areas under curve.
