# Supplementary figures and images for: Comprehensive Analysis of Senescence Characteristics Defines a Novel Prognostic Signature to Guide Personalized Treatment for Clear Cell Renal Cell Carcinoma
Source: Front Immunol. 2022 Jun 2;13:901671. doi: 10.3389/fimmu.2022.901671 (PMC9201070; doi:10.3389/fimmu.2022.901671)

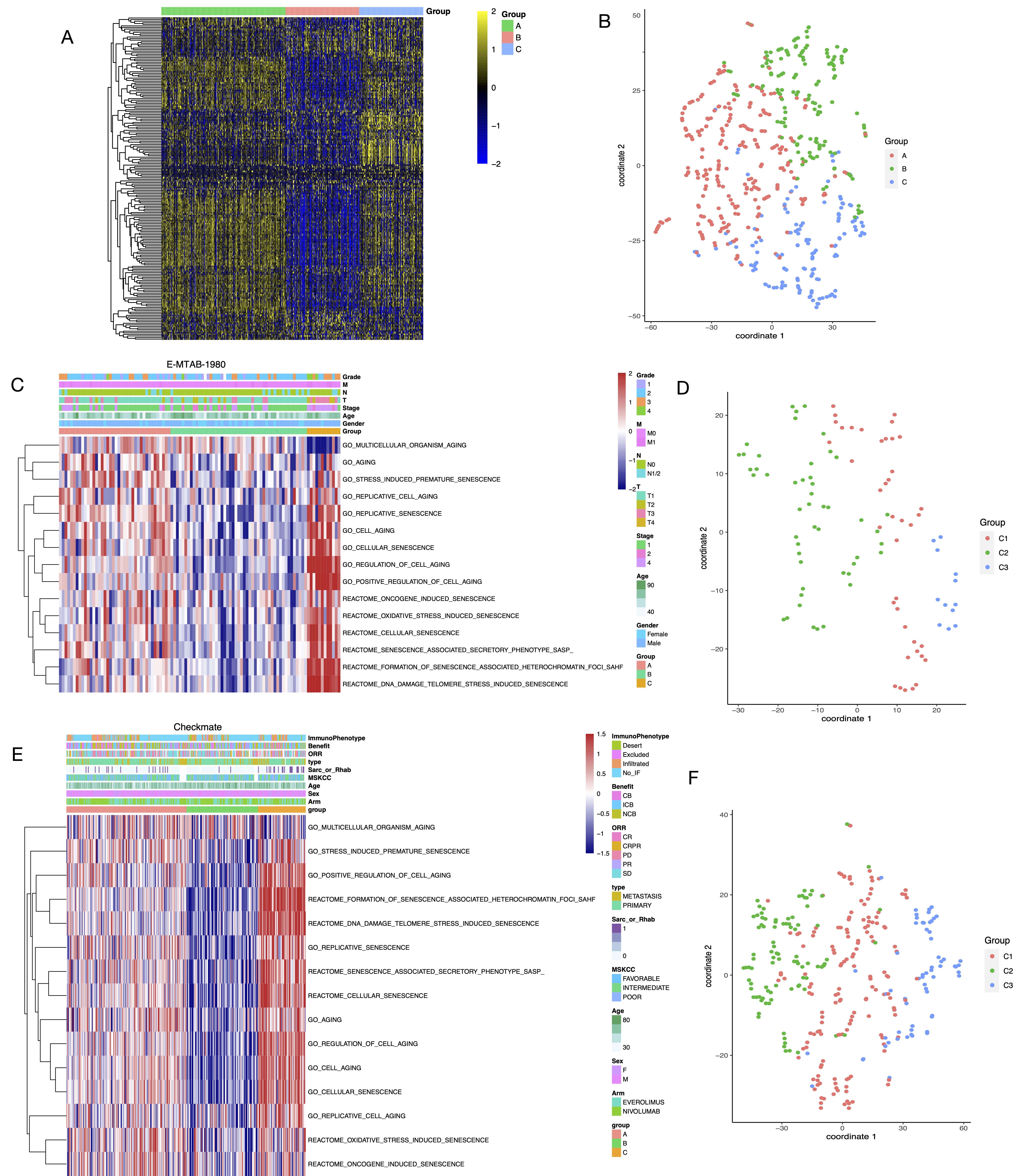

Supplement: Supplementary Figure 1 — Regeneration of the senescence subtypes in external cohorts. (A) Heatmap displayed the expression level of the DESGs across the senescence subtypes in TCGA-KIRC. (B, D, F) the t-SNE down-dimension plots showed good discrimination of tumor samples among the senescence subtypes in TCGA-KIRC (B), E-MTAB-1980 (D), and Checkmate (F). (C, E) Heatmaps of the 15 senescence-related biological processes in E-MTAB-1980 (C) and Checkmate (E). [file Image_1.jpeg]

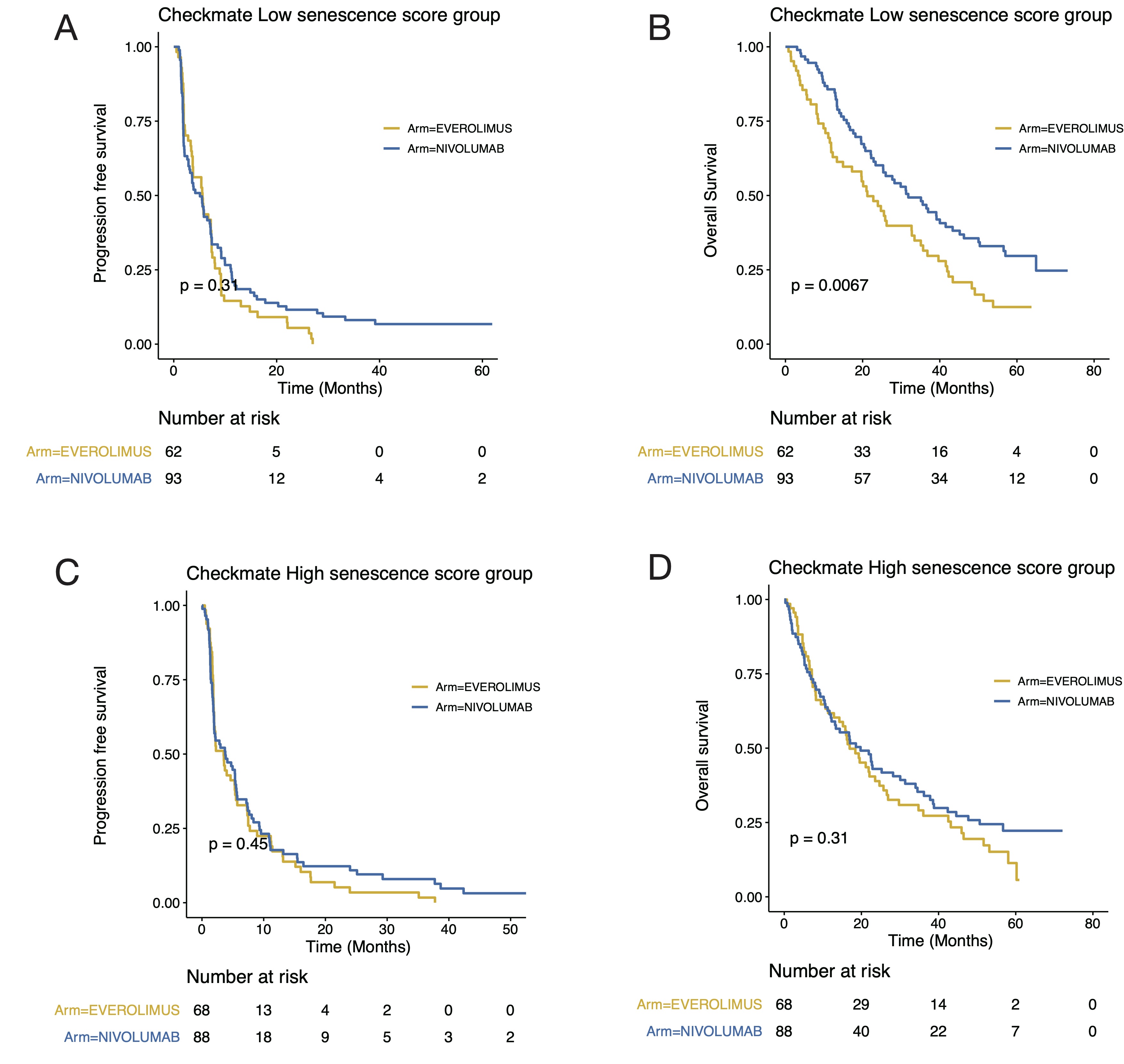

Supplement: Supplementary Figure 2 — Subgroup analysis of ICB treatment benefit versus targeted therapy in Checkmate. (A, B) In patients of the low-senescence score group, the use of Nivolumab resulted in an OS, but not a PFS benefit over Everolimus. (C, D) In the high-senescence score group, there was no PFS or OS difference between the Nivolumab- and Everolimus-treatment arms. [file Image_2.jpeg]
